# Emotional real-world scenes impact visual search

**DOI:** 10.1007/s10339-018-0898-x

**Published:** 2018-12-24

**Authors:** Robert C. A. Bendall, Aisha Mohamed, Catherine Thompson

**Affiliations:** Directorate of Psychology and Public Health, School of Health Sciences, University of Salford, Salford, M5 4WT UK

**Keywords:** Emotion, Cognition, Visual attention, Visual search, Real-world scenes, Bayesian analysis

## Abstract

Research shows that emotional stimuli can capture attention, and this can benefit or impair performance, depending on the characteristics of a task. Additionally, whilst some findings show that attention expands under positive conditions, others show that emotion has no influence on the broadening of attention. The current study investigated whether emotional real-world scenes influence attention in a visual search task. Participants were asked to identify a target letter embedded in the centre or periphery of emotional images. Identification accuracy was lower in positive images compared to neutral images, and response times were slower in negative images. This suggests that real-world emotional stimuli have a distracting effect on visual attention and search. There was no evidence that emotional images influenced the spatial spread of attention. Instead, it is suggested that findings may provide support for the argument that positive emotion encourages a global processing style and negative emotion promotes local processing.

**Electronic supplementary material:**

The online version of this article (10.1007/s10339-018-0898-x) contains supplementary material, which is available to authorized users.

## Introduction

Selective visual attention refers to the biasing of attentional resources due to an inability to attend to all items and areas of the visual world simultaneously. This biasing of resources is dependent upon top-down processing (characterised by goal-directed behaviour, e.g. searching for a target item located in a visual display) and bottom-up processing (characterised by automatic capture of attention by salient information in the environment regardless of task demand, e.g. Itti and Koch 2000). When presented with emotional information, or during an emotional situation, these two competing processing strategies are also evident. For instance, emotion influences cognition in a bottom-up manner, whilst simultaneously, individuals adopt top-down cognitive control strategies to direct resources to emotion regulation (Ochsner et al. 2012). Consequently, emotion has the potential to influence resources and impact selective attention.

It has recently been suggested that emotional stimuli may provide an additional type of influence on attentional processing and selective attention beyond the traditional top-down and bottom-up distinctions (Pourtois et al. 2013). This influence has been referred to as emotional attention (Vuilleumier 2005), and research has suggested that top-down processing, bottom-up processing, and emotional processing can have a summative impact on selective attention (Brosch et al. 2011). It is proposed that these three systems can operate simultaneously and are able to exert separate influences upon visual attention (Pourtois et al. 2013).

A number of recent review articles have demonstrated the ability of both negative stimuli (e.g. Carretié 2014; Pourtois et al. 2013) and positive stimuli (e.g. Carretié 2014; Pool et al. 2016) to impact selective attention. This is evidenced by more accurate and faster detection of emotional targets in a variety of tasks (e.g. during visual search; Eastwood et al. 2003; Williams et al. 2005), as well as by the increased ability of emotional distractors to capture attention compared to neutral distractors (Carretié 2014). Initial research in this area has focused on the investigation of the negative emotion fear, which is often operationalised using threat-related stimuli (Carretié 2014; Eastwood et al. 2003; Pourtois et al. 2013; Williams et al. 2005).

The majority of the research in this field has adopted stimuli depicting emotional faces or employed tasks using stimuli arrays incorporating emotional targets and/or distracters, and little research has made use of more naturalistic stimuli or visual scenes. One study that did employ natural scenes demonstrated that search performance was reduced for negative stimuli compared to neutral stimuli (Simpson et al. 2000). Using natural real-world images from the International Affective Picture System (Lang et al. 2008), Simpson et al. found that when participants were asked to make a judgement regarding the number of humans located within a visual scene response times increased when the scene was negative compared to neutral. This finding demonstrates that the emotional content of natural scenes has an influence on visual attention.

A predominant theory to account for the influence of emotion on attention is the broaden-and-build theory (Fredrickson 2001) which proposes that positive emotions (e.g. joy, interest, contentment, love) have the ability to “broaden” an individual’s “thought-action repertoires” and “build” an individual’s “enduring resources” (Fredrickson 2001, p. 218). The theory also suggests that negative emotion (e.g. anxiety, sadness, anger, despair) has the opposite effect, preventing an individual from thinking broadly and building lasting psychological reserves. Initial support for the broadening influence of positive emotion on attention (and therefore the broaden-and-build theory) comes from studies that have utilised the global–local processing task (Navon 1977). These early studies demonstrated that positive emotion promotes a global processing style, whereas negative emotion encourages local processing, and researchers argue that this shows that positive emotions “expand” attentional resources, allowing individuals to process more information (Basso et al. 1996; Fredrickson and Branigan 2005). However, these initial studies do not specifically measure the spread (and capacity) of attention, and rather suggest that emotion may influence processing style (a preference towards local or global processing) without affecting the attentional resources available (Bendall and Thompson 2015; Taylor et al. 2017).

This argument is consistent with the levels-of-focus hypothesis (Clore et al. 2001) which proposes that emotions guide processing whereby positive mood encourages a focus on the most accessible information within the mind, whereas negative mood will lead to more emphasis on external, incoming information. Given that global processing is proposed to be the default method of information processing (Fiske and Taylor 1991), it is theorised that positive emotions will allow greater focus on internal, accessible information, and will therefore be more likely to activate a global strategy compared to negative emotions. Gasper and Clore (2002) provided evidence to support this theory, showing that when induced into a negative mood, participants were less likely to focus resources at a global level. They argue that the findings provide no support for differences in the amount of resources or the amount of processing taking place, and instead emotion influences the level at which resources are focused.

More recently, different tasks have been used to investigate the influence of emotion on attention. Wadlinger and Isaacowitz (2006) presented individuals with three images simultaneously and used eye-tracking to measure the allocation of attention. One image was located in the centre of the screen and two were located in the periphery. Participants who were induced into a positive emotional state made a greater number of fixations on peripheral stimuli than those individuals induced into a neutral emotional state. Wadlinger and Isaacowitz (2006) suggest that this shows the expansion (or broadening) of visual attention under conditions of positive emotion. Rowe, Hirsh, and Anderson (2007) also provide findings to support a broadening effect under conditions of positive emotion. Using a modified version of the Eriksen flanker task (Eriksen and Eriksen 1974), they compared the influence of near and far peripheral distractions under positive, neutral, and negative moods. Participants induced into positive emotion were more distracted by the far flankers, again suggesting that during positive emotion attention expands and allows an individual to process additional information. Despite the fact that in these studies the manipulation of emotion was the emotional state of the observer, rather than the emotional content of the scene, these findings are consistent with those from Simpson et al. (2000) showing that emotion affects attention and search.

Further studies have failed to show a broadening of visual attention as a result of emotion. For example, using a change detection task that specifically measured the allocation of attention to central and peripheral information (allowing comparison of attention to the centre and periphery of a natural visual scene), Bendall and Thompson (2015) found no influence of emotion on attention using real-world stimuli. However, the stimuli in the change detection task were controlled so that only those of neutral valence were used and this may have influenced the results. Prior to completing this task participants were induced into positive, negative, and neutral mood states, yet as the change detection trials then involved neutral stimuli the mood induced may have reverted to neutral.

The research discussed thus far demonstrates two different approaches to the investigation of emotional influences on visual attention. A number of studies have focused on the effect of emotional state on attention (e.g. Basso et al. 1996; Bendall and Thompson 2015; Bruyneel et al. 2013; Fredrickson and Branigan 2005; Gasper and Clore 2002; Rowe et al. 2007; Wadlinger and Isaacowitz 2006), whilst other research has investigated the impact of emotional stimuli on attention (Carretié 2014; Eastwood et al. 2003; Pool et al. 2016; Pourtois et al. 2013; Simpson et al. 2000; Vuilleumier 2005; Williams et al. 2005). This study focuses on the latter, but rather than measuring attentional capture of, or attentional orienting towards emotional stimuli, it investigates the effects of emotional scenes on the ability to identify neutral targets.

In general, research indicates that when the emotional content of stimuli is varied during visual search tasks positive and negative information can have similar influences on attention. However, the majority of these studies have used emotional distractors (see Carretié 2014), artificial displays (Eastwood et al. 2003), or emotional targets (Eastwood et al. 2003; Williams et al. 2005). Consequently, it is not currently known how positive and negative real-world scenes impact visual search towards neutral targets, and this is one of the aims of the current study. One advantage of adopting real-world scenes is that they more closely resemble the everyday environment and therefore provide increased ecological validity. In the current study, a modified version of the visual search task designed by Brockmole and Henderson (2006) was used to measure the effect of emotional real-world scenes on visual attention. Participants were asked to identify targets embedded in images of varying emotional valence (neutral, negative, and positive) from the Nencki Affective Picture System (NAPS; Marchewka et al. 2014). The NAPS is a large collection of real-world images that have each been rated for valence to allow for the precise control and manipulation of experimental stimuli. Previous literature has focussed on the ability of negative emotional stimuli to influence visual attention (see Carretié 2014), and it has been argued that research investigating the impact of emotional stimuli on visual attention needs to include a positive emotion condition to allow more precise conclusions to be reached (Bendall et al. 2016; Carretié 2014). As a result, three experimental conditions were included in the current experiment: neutral stimuli, negative stimuli, and positive stimuli. The study design allowed the investigation of whether emotional real-world stimuli impact visual search to neutral targets. Research investigating the emotional modulation of attention during visual search tasks incorporating neutral targets is limited and was an additional aim of the current investigation. It is important to adopt visual search paradigms that include neutral targets as this reflects instances in which individuals are required to attend to neutral targets within an emotional situation. It was predicted that identification of search targets embedded within positive and negative stimuli would be less accurate and slower compared to targets embedded within neutral stimuli. Additionally, as the targets were located in the centre or periphery of each image, this also provided a measurement of any “broadening” or “narrowing” of attention. If positive information does have a broadening effect on visual attention, this will increase the breadth of attention and therefore the amount of information an individual can process. Consequently, it is predicted that peripheral targets will be identified significantly quicker and more accurately when embedded in positive images compared to neutral images.

## Method

### Participants

Based on previous studies, this experiment aimed for a minimum sample size of 36, and in total 39 female participants completed the experiment. Participants were an opportunity sample of students from the University of Salford aged between 18 and 37 years (M = 22.69, SD = 4.12). Where appropriate, volunteers received course credit for participating. Ethical approval was obtained from the School of Health Sciences & School of Nursing, Midwifery, Social Work and Social Science Ethics Approval Committee at the University of Salford.

### Design

A within-participants design was used with two independent variables; *location* of the search target in the visual search task (central or peripheral) and *emotional stimuli* used during this task (positive, neutral, or negative). The dependent variables consisted of accuracy (percentage correct) and response time (in seconds) to identify the search target.

### Materials

The experiment was designed and run using E-Prime (Psychological Software Tools, Inc.) and participants completed the study using a Viglen Intel i7 Core computer with a 60 Hz, 22 in. monitor. Images from the NAPS (Marchewka et al. 2014) consisting of objects, landscapes, people, and animals were selected on the basis of their affective valence ratings, and a total of 192 images were used for the visual search task: 64 positive, 64 neutral, and 64 negative images (identification numbers are provided in Electronic Supplementary Material 1). The NAPS is a database of real-world scenes, and each image has been rated by 204 individuals to provide a valence rating. All images in the visual search task were presented in colour and measured 1600 × 1200 pixels. In each trial, a target (the letter T or the letter L) was presented over the image. The targets were shown in Arial font size 12 in blue and were presented at 0°, 90°, 180°, or 270° orientations. Central targets were located within the centre of the image within an area measuring 450 × 342 pixels, and peripheral targets were located outside of this area.

The NAPS database provides the physical properties of each image including a measure of image complexity indexed using image JPEG size, entropy, luminance, and contrast. It was important to check that the stimuli sets differed in valence but were similar in these physical characteristics. All statistical analyses were conducted using JASP adopting a Bayesian approach (JASP Team 2017). JASP calculates Bayes factors (BF) on distributions of effect size to assess the relative probability of observed data between two competing statistical hypotheses; the null hypothesis (H0) and the alternative hypothesis (H1) (see Jarosz and Wiley 2014 for an introduction to Bayesian statistics). BFs are reported expressing the probability of the data given H1 relative to H0 where values larger than 1 represent evidence for H1. To investigate the evidence for and against an effect of valence within our stimuli sets, a Bayesian one-way repeated measures analysis of variance (ANOVA) with default priors selected was completed. This result supported the predicted difference in valence scores between the three stimuli sets (positive, neutral, and negative). Analysis revealed a BF_10_ of 4.398e+138 in favour of the alternative hypothesis suggesting that the data are 4.398e 138 times more likely to be observed under the alternative hypothesis (Table 1). A BF of greater than 100 is considered extreme evidence for the alternative hypothesis (Jeffreys 1961). Post hoc analysis revealed a BF_10_ of 1.012e+52 for the comparison between positive stimuli (M = 7.70, SD = .34) and neutral stimuli (M = 5.01, SD = .20) demonstrating higher valence scores in the positive stimuli set compared to the neutral stimuli set. The comparison between positive stimuli and negative stimuli (M = 2.55, SD = .58) provided a BF_10_ of 5.150e+56 in favour of the alternative hypothesis suggesting that valence scores in the positive stimuli set were greater than the negative stimuli set. Finally, the comparison between neutral stimuli and negative stimuli revealed a BF_10_ of 9.090e+36 in favour of the alternative hypotheses suggesting that valence scores were greater in the neutral stimuli set compared to the negative stimuli set. A series of Bayesian one-way repeated measures ANOVAs with default priors selected were also completed to investigate if the experimental stimuli differed across valence conditions for image complexity, entropy, luminance, and contrast. BFs_10_ of .375, .086, .067, and .104 were produced for complexity, entropy, contrast, and luminance, respectively, supporting the null hypotheses. These findings demonstrate that the stimuli sets did not differ in levels of these characteristics (Table 1). It should, however, be noted that the stimuli sets differed with regard to levels of arousal (Table 1; see “Discussion” section for further details). A Bayesian repeated measures ANOVA produced a BF_10_ of 1.920e+57 providing extreme support for the alternative hypothesis. Post hoc analyses for arousal revealed BF_10_s of 1.609e+19, 7.207e+29, and 1.599e+19 for negative–neutral, negative–positive, and neutral–positive comparisons, respectively.

**Table 1:** Physical characteristics of experimental stimuli

Stimuli set	Valence		Complexity		Entropy		Contrast		Luminance		Arousal	
	M	SD	M	SD	M	SD	M	SD	M	SD	M	SD
Positive	7.70	.34	331,574	112,027	7.46	.38	62.60	13.14	120.55	30.47	3.96	1.01
Neutral	5.01	.20	333,836	120,486	7.50	.36	65.10	13.92	118.64	33.11	5.19	.60
Negative	2.55	.58	369,031	128,968	7.52	.38	62.82	12.22	117.01	29.60	6.69	.66

*M* mean, *SD* standard deviation, *N* = 64

### Procedure

After providing written informed consent, participants were seated approximately 22 inches from the screen and given full instructions about the task. If they were happy to proceed with the experiment, they pressed the spacebar and were presented with an example negative image for 5 s to allow consideration of whether to continue with the experiment. Following this, they were shown onscreen instructions as a reminder of what the task entailed and were asked to press the spacebar when ready to begin. In each trial, a fixation cross was presented for 1000 ms following which an image was presented with the letter T or L superimposed. The image remained on the screen until participants had identified the target as a T or L by pressing the corresponding key. If they were unable to locate or identify the target, they had the option of pressing the spacebar to terminate the trial but were asked to use this as a last resort. Once a response had been made, feedback was provided onscreen for 500 ms and following this the next trial began. Participants completed a total of 192 trials presented in a random order. There were 64 images for each condition of valence, half showing the letter T and half showing the letter L. In these 32 images, the target was presented at a randomly selected central location for 16 trials and a randomly selected peripheral location for 16 trials. There were 4 trials for each angle of rotation for each condition. After completing the visual search task, participants were asked to view 10 positive images from the NAPS (mean valence 8.20) for 2 s each. This was to ensure that they were not adversely affected by the negative images when leaving the laboratory.

## Results

Data collected included accuracy (percentage correct; %) and response times (seconds; s) to the visual search task (raw data are provided in Electronic Supplementary Material 2). Overall accuracy was 95.05%, and participants took an average of 1.72 s to correctly locate a target. A total of 74 trials were terminated (0.01% of trials), and the number of terminated trials did not differ according to stimuli valence (Electronic Supplementary Material 3). A further 116 trials (2.48% of total correct trials) were removed at ± 2 standard deviations from the mean on the basis of reaction time. Two participants were excluded from the analysis due to mean accuracy that was ± 2 standard deviations from the mean. A further two participants were removed because their mean response times were ± 2 standard deviations from the mean.

**Fig. 1:**
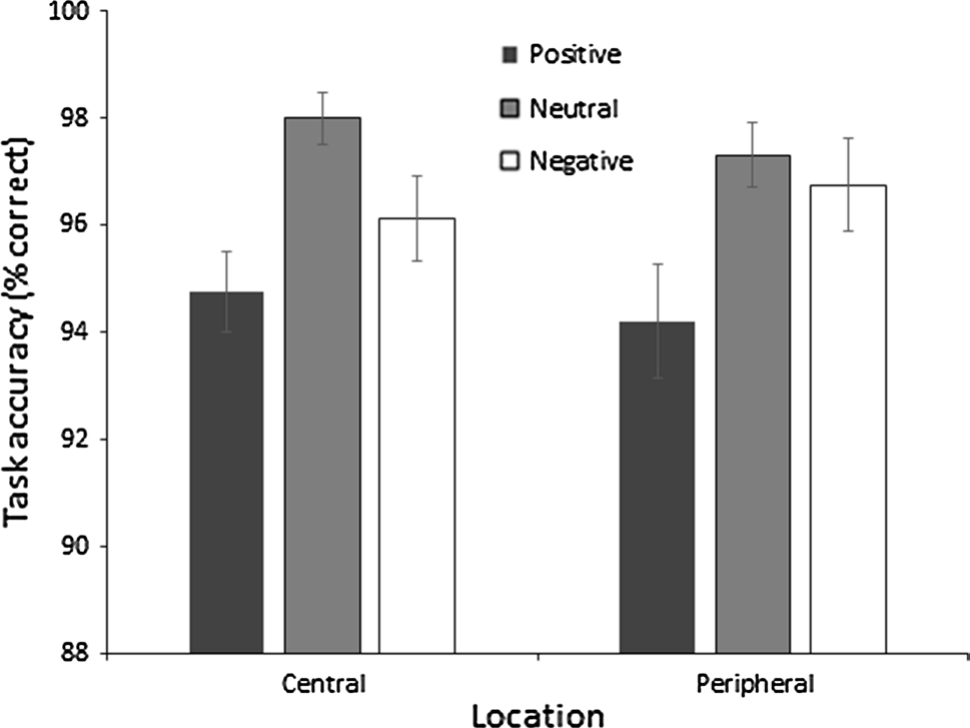
Mean percentage accuracy in the visual search task. Error bars = standard error mean

Two 2 (*location; central or peripheral*) × 3 (*stimuli valence; positive, neutral, or negative*) Bayesian repeated measures ANOVAs with default priors selected were completed followed by post hoc comparisons. Analysis of accuracy revealed that the model including only valence outperformed all other models where a BF_10_ of 759.763 was observed in support of H1 suggesting that the data are 759.763 times more likely to be observed under the alternative hypothesis. This suggests that there is extreme evidence in favour of H1 where the valence of the stimuli had an effect on accuracy scores in the visual search task. Post hoc analyses for valence revealed BF_10_s of 1.822, 3.604, and 260.585 for negative–neutral, negative–positive, and neutral–positive comparisons, respectively. These observations suggest that there was anecdotal evidence for H1 in the negative–neutral comparison, moderate evidence in support of H1 for the negative–positive comparison, and extreme evidence in support of H1 for the neutral–positive comparison. These findings suggest that accuracy in the visual search task was comparable for negative and neutral stimuli, but that accuracy was reduced for positive stimuli compared to neutral stimuli. Additionally, there was moderate evidence to suggest that accuracy was also reduced for positive stimuli compared to negative stimuli (Fig. 1). For location, a BF_10_ of .190 was observed indicating anecdotal evidence in support of H0 suggesting that location had no impact on accuracy in the visual search task. Finally, the interaction effect model produced a BF_10_ of .107 (15.869/148.189) indicating substantial evidence against an interaction between location and valence.

**Fig. 2:**
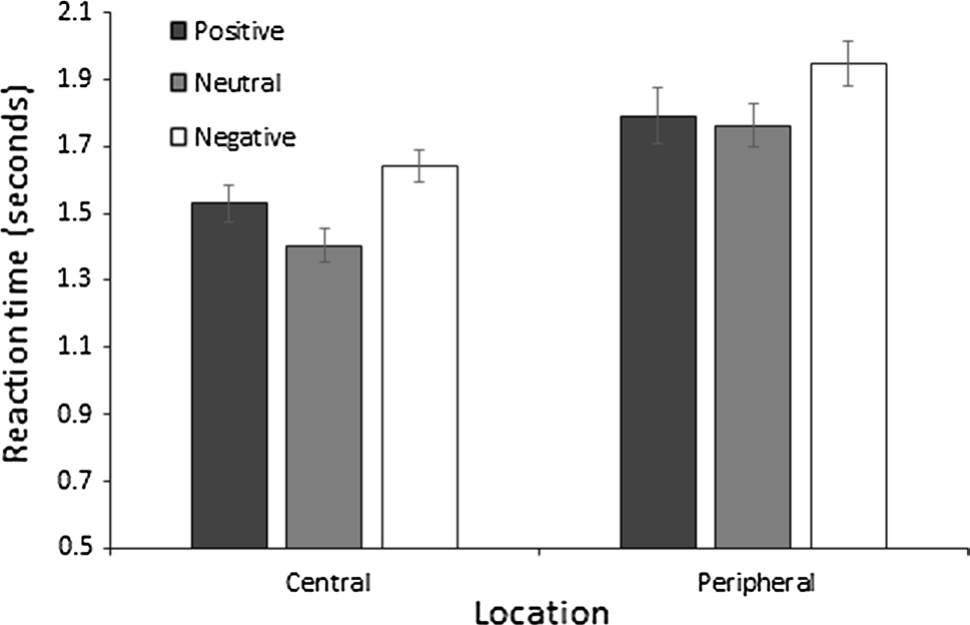
Mean response time to correctly detect search targets. Error bars = standard error mean

Analysis of reaction time revealed that the model including valence and location outperformed all other models where a BF_10_ of 1.573e+11 was observed providing extreme support for H1. A BF_10_ of 19.949 was observed for valence indicating strong evidence for H1. Post hoc analyses for valence revealed BF_10_s of 502.520, 2.004, and .501 for negative–neutral, negative–positive, and neutral–positive comparisons, respectively. These observations suggest that there was extreme evidence for H1 in the negative–neutral comparison where reaction time in the visual search task was quicker for neutral trials compared to negative trials. Anecdotal evidence was observed in support of H1 for the negative–positive comparison, whilst there was anecdotal evidence in support of H0 for the neutral–positive comparison, suggesting that reaction time in the visual search task did not differ between negative and positive trials and between neutral and positive trials (Fig. 2). For location, a BF_10_ of 1.375e+9 was observed indicting extreme evidence for H1 suggesting that central targets were identified quicker than peripheral targets. Finally, the interaction model produced a BF_10_ of .118 (1.849e+10/1.573e+11) indicating substantial evidence against an interaction between location and valence.

## Discussion

The aim of the current study was to investigate the allocation of attention to neutral targets within emotional real-world scenes. The experiment was designed to measure whether emotional stimuli affect visual search task performance. Additionally, the study investigated whether positive emotional stimuli have the potential to broaden visual attention and whether negative emotional stimuli can narrow attention. This was investigated by adopting a simple visual search task in which neutral targets were situated in the centre or periphery of positive, negative, and neutral images and participants had to identify each target as quickly and as accurately as possible.

Participants were quicker to detect visual search targets when they were presented centrally compared to in the periphery. This main effect is important as it endorses the design of the visual search task. To establish any influence of emotional stimuli on the allocation of visual attention to central and peripheral locations, it was important to initially demonstrate that visual attention and search followed the expected pattern, with resources located to the centre of a scene before the periphery during control conditions (neutral stimuli). Differences in response times to detect central and peripheral targets have been reported previously in studies investigating visual attention (Bendall and Thompson 2015; Tatler 2007). For instance, during a change detection flicker task, where participants are presented with two images separated by a brief inter-stimulus-interval and are required to identify a change in one of the images, Bendall and Thompson (2015) found that changes made to the centre of a visual scene were located faster than changes made in the periphery of a visual scene. The current findings support this past research.

In addition to showing that attention is allocated to information at the centre of a scene before being allocated to the periphery, there was also evidence that attention varied according to the emotional valence of the stimuli. Participants were less accurate at identifying the target when it was presented in a positive image compared to a neutral image. It also took participants longer to correctly identify a target when it appeared on a negative image compared to a neutral image. These findings demonstrate that both positive real-world stimuli and negative real-world stimuli can influence visual attention, but suggests that they may do so in different ways. In accordance with the levels-of-focus hypothesis proposed by Clore et al. (2001), we suggest that emotional information may influence processing style whereby positive stimuli promotes a global processing style, and negative stimuli encourages a local processing style. This theory proposes that positive moods encourage greater focus on internal, accessible information (with a global processing strategy being the most “usual” and therefore accessible method of processing) and negative moods prevent this internal focus and instead lead to more local processing of external, incoming information. In the current study, the reduced accuracy to identify targets in positive images (with no impact on response times) is indicative of impaired *identification* of the search target. This suggests that participants were allocating attention to the wider scene (global processing) so could locate the target, but could not accurately identify this due to a lack of attention at the local level. Conversely, longer response times to identify targets in negative images (with no impact on accuracy) suggest that *localisation* of the target may be impaired. It may be argued that it took participants longer to find the target in negative images due to a lack of attention at the global level, but when they did find the target they could identify it with a high level of accuracy.

Whilst it is proposed that the findings provide support for the levels-of-processing hypothesis, this interpretation cannot be tested with the current experimental design. The dependent variables measured the ability to identify the target, and future work should attempt to separate out detection (localisation) and identification. In addition, the experimental design is substantially different from other studies intending to test this hypothesis. For instance, Gasper and Clore (2002) induced participants into positive or negative mood states and asked them to try and interpret a drawing before reproducing it from memory. They found that participants in a positive mood were more likely to reproduce the global features within the drawing compared to those in a negative mood. The accuracy and extent of recall did not vary across the mood conditions, again offering no support for the proposal that mood affects the amount of resources available for processing. It should be noted that the levels-of-focus hypothesis posits that mood affects attention and processing when it is relevant to the task as the emotional cues are processed as information pertaining to the task and this biases the individual to a global or local focus. In the current study, the valence of the scene has no relevance to the task itself and the targets are neutral, yet an effect of valence is still apparent. By using real-world scenes, it is possible to show that even when individuals are not experiencing a particular mood and they are not required to attend to the emotional content of a situation, their attention may still be influenced by that content and the information within the situation may cue a particular processing style.

Simpson et al. (2000) also used real-world scenes to investigate the influence of emotional stimuli on visual search. In their experimental task, participants had to count the number of individuals present in a scene and this could either be one person or two. Findings showed that response times in the visual search task were slower for negative stimuli compared to neutral stimuli. These findings are replicated in the current work, with slower response times to targets presented in negative scenes. Additionally, Simpson et al. (2000) found a significant interaction between stimuli valence and trial type for response accuracy. Increased accuracy was evident for negative trials compared to neutral trials when the correct target response was “two”, whereas accuracy was reduced for negative trials compared to neutral trials when the correct response was “one”. Whilst our findings showed no effect of negative stimuli on accuracy, it may be argued that the findings of Simpson et al. lend support to the proposal that negative information encourages a local style of processing. In that study, participants knew there was a maximum of two possible targets, and when two targets have been located the search has been exhausted. At this point, the use of local processing (in the negative condition) means the target is identified more accurately. However, when there is only one search target it is possible that participants continue to search and accuracy suffers as a result because participants do not benefit from this bias towards local processing.

Whilst showing that attention is allocated to information in the centre of the scene before being allocated to the periphery, the lack of any interaction between target location and emotion shows no evidence of a broadening or narrowing of visual attention due to emotion. This provides no support for the broaden-and-build theory, which suggests that emotion has the ability to affect processing resources and modulate the scope of attention. The research utilising tasks that measure the spatial allocation of attention (e.g. Bendall and Thompson 2015) show limited evidence for any narrowing or broadening of attention due to emotion. The current findings suggest that whilst emotional stimuli can influence the manner in which an individual will process visual information, it does not necessarily influence the amount of resources available to process this information. We argue that because many previous studies adopt tasks such as the global–local processing task, they are unable to directly measure the spatial allocation of visual attention. Instead of measuring the breadth of visual attention, a number of paradigms have measured processing strategy. Further research is needed to explicitly test this argument, and this is an avenue for future work.

Two limitations of the current study are worthy of note, and these could be rectified in future experiments. Firstly, our sample was entirely female, and so it is plausible that the current findings may not be generalisable to males. For instance, sex differences have been observed in self-report ratings of negative affect and fronto-limbic connectivity during emotional processing (Lungu et al. 2015) when attending to disgust facial expressions (Kraines et al. 2017) and during response inhibition in emotional contexts (Ramos-Loyo et al. 2016). Further research is needed to investigate if the observed effects of emotional stimuli reported here in females are also evident for males. Secondly, our experimental stimuli sets, whilst controlled for image complexity, entropy, contrast, and luminance, differed in levels of arousal. Valence is often linked to arousal where negatively valenced stimuli are highly arousing whilst positively valenced images are rated as low in arousal. Therefore, we cannot reject the assertion that the observed effects were due to alterations in stimuli arousal rather than manipulations in valence. Whilst this is an avenue for future research, the current findings still demonstrate that emotional stimuli (whether in terms of valence or arousal) have an impact on visual search performance.

Studies show differing effects of emotion on attention, and this is influenced by a range of factors such as the nature of the task and the characteristics of the emotions themselves. The current study used a visual search task incorporating real-world stimuli to investigate the effect of emotion and found that emotional stimuli distracted attention during visual search. This finding adds to the literature suggesting that emotional stimuli capture attention in visual search tasks using abstract arrays and/or emotional targets and provides evidence that this finding extends to visual search tasks using emotional real-world images and neutral targets. Additionally, due to the different effects of positive emotional stimuli and negative emotional stimuli on reaction time and response accuracy, there is some evidence to support the concept that positive information encourages a more global processing style, and negative information promotes a more local processing style. However, there was no evidence for a broadening of attention for positive emotional images or a narrowing of attention for negative emotional images. The current results provide further evidence for the complex relationship between emotion and visual attention. The research supports empirical findings in the literature and indicates that emotion does have an impact on the way in which attentional resources are allocated.

## Electronic supplementary material

Below is the link to the electronic supplementary material.

Supplementary material 1 (XLSX 11 kb)

Supplementary material 2 (DOCX 13 kb)

Supplementary material 3 (XLSX 15 kb)
