# Supplementary material for: Emotional real-world scenes impact visual search
Source: Cogn Process. 2018 Dec 24;20(3):309–16. doi: 10.1007/s10339-018-0898-x (PMC6647383; doi:10.1007/s10339-018-0898-x)
Supplement: Supplementary file 3 — Supplementary material 3 (XLSX 15 kb) [file 10339_2018_898_MOESM3_ESM.docx]

Supplementary results - Bayesian analysis of terminated trials

Table 1

| *Terminated trials* |  |  |  |  |  |  |  |  |  |  |
| --- | --- | --- | --- | --- | --- | --- | --- | --- | --- | --- |
| Stimuli set | *M* | *SD* |  |  |  |  |  |  |  |  |
| Positive | .70 | 1.69 |  |  |  |  |  |  |  |  |
| Neutral | .27 | 1.20 |  |  |  |  |  |  |  |  |
| Negative | .55 | 1.98 |  |  |  |  |  |  |  |  |

*M* = mean, *SD* = standard deviation, *N* = 64

A Bayesian repeated measures ANOVA produced a BF_10_ of .902 providing no support for the experimental hypothesis. These findings demonstrate that trials were terminated to a similar degree irrespective of their valence.
